# New Appliances & Things Medical

**Published:** 1911-08-26

**Authors:** 


### NEW APPLIANCES & THINGS MEDICAL.

### ADALIN.

The Bayer Company, Ltd., 19 St. Dunstan's Hill, E.C.

Adalin is bromo-di-ethyl-acotyl-urea, and is used as a new sedative and hypnotic. It is a white powder, insoluble in cold, but easily soluble in hot water, which has been used as an adjuvant to general and local anæsthesia, and its harmlessness and the facility with which it can be prescribed are great points in its favour. It appears to us to be one of the most useful sedatives put on the market lately, and practitioners who are anxious to try it should write for the literature and a free sample, which will be forwarded on application to the manufacturers.

### INSTITUTIONAL BEDDING.

The Lancashire Bedding Company, Ltd., Marlborough Street, Vauxhall Road, Liverpool.

Mr. Golightly's catalogue contains a large number of interesting models of wire, hair, and fibre mattresses, bolsters and pillows, and the prices shown compare very favourably with those of other makers. When we take into consideration the fact that the Lancashire Bedding Company's goods may be relied upon to be thoroughly of excellent make and admirable quality, some of the prices charged seem ridiculously low. For instance, the firm's institutional hair and fibre three-inch border mattress (3 feet H.F. 3 quality) is listed at 18s., an astonishingly low price when the durability of the article is taken into consideration. We have had an opportunity of trying this mattress under exceptionally severe conditions in an outdoor shelter, and have found it eminently satisfactory. The firm specialises in hospital, hotel, and institutional mattresses, and hospital secretaries who have not yet tried its models and compared them with others should write for its price lists and at least experiment with H.F. 3.

